# Advances in human microbiome and prostate cancer research

**DOI:** 10.3389/fimmu.2025.1576679

**Published:** 2025-04-14

**Authors:** Xin Pei, Lei Liu, Yuping Han

**Affiliations:** ^1^ Department of Urology, China-Japan Union Hospital, Jilin University, Changchun, China; ^2^ Department of Endocrine, China-Japan Union Hospital, Jilin University, Changchun, China

**Keywords:** prostate cancer, urinary microbiome, gut-prostate axis, tumor microenvironment, microbiome

## Abstract

Prostate cancer (PCa) is the second most common malignant tumor in men worldwide, and its metastatic and heterogeneous nature makes it significantly more difficult to treat. Recent studies have revealed the critical role of microbiota in PCa occurrence, progression, and treatment. Accumulating evidence from 16S rRNA and metagenomic sequencing suggests the presence of specific microbiota in prostate tissues and macrogenomics techniques: cancerous tissues are enriched with pro-inflammatory genera (e.g., *Fusobacterium*, *Propionibacterium acnes*), whereas commensal bacteria (e.g., *Pseudomonas*) are more common in paracancerous tissues. The microbiota drive tumor progression through activation of the NF-κB/STAT3 pathway to induce chronic inflammation, modulation of the immune microenvironment (e.g., Treg/Th17 imbalance and M2-type macrophage polarization), and metabolite (e.g., LPS, short-chain fatty acids)–mediated hormonal and epigenetic regulation. In terms of clinical translation, urinary microbiota characterization combined with metabolomics analysis may enhance diagnostic specificity, while gut flora modulation (e.g., probiotic interventions or fecal transplants) may improve resistance to androgen deprivation therapy. Current challenges include sequencing accuracy of low-biomass samples, limitations of causal mechanism validation models, and large cohort heterogeneity. In the future, it will be necessary to integrate multi-omics technologies to explore the bidirectional regulation of the “gut-prostate axis” and develop personalized therapeutic strategies targeting microorganisms. In this paper, we systematically review the interactions between microbiota and PCa and their clinical potentials to provide a theoretical basis for precision diagnosis and treatment.

## 1 Introduction

According to GLOBOCAN data in 2023, prostate cancer (PCa) ranks as the second most common cancer incidence and fifth most common mortality in men worldwide, and is high on the list of urologic tumors (1). Clinical staging includes both limited (early) and metastatic (advanced) PCa, with early stage patients having a high 5-year survival rate, but approximately one-third progressing to castration-resistant prostate cancer (CRPC), leading to a significant increase in therapeutic difficulty. The therapeutic challenge stems mainly from the heterogeneity of metastatic prostate cancer, drug resistance, and the lack of effective biomarkers, especially at the CRPC stage, where patient quality of life and prognosis are extremely poor (2). The microbiome refers to the collection of commensal microorganisms in the host, including bacteria, viruses, fungi, and so forth, whose composition and function can be studied by techniques such as 16S rRNA sequencing (sequence analysis based on conserved bacterial genes) and macrogenomics (whole genome sequencing) 16S rRNA sequencing identifies bacterial species by amplifying specific gene regions, whereas macrogenomics comprehensively analyzes the gene function of the microbial community (3) (4). For example, several studies have utilized these technologies to discover microbiome specificities in PCa patients, such as the enrichment of Propionibacterium acnes (5). Traditionally, the prostate has been considered a sterile organ, but recent studies have demonstrated the presence of a microbiome in the prostate by high-sensitivity sequencing and that its composition is associated with prostate disease (6).

## 2 Differences in the microbiome of prostate tissue

### 2.1 Characterization of the microbiome of the healthy prostate gland

The microbiome of the healthy prostate is dominated by the genera Cutibacterium (e.g., *C. acnes*), Escherichia (Escherichia coli), Pseudomonas, and Staphylococcus , which constitute its core microbial community. Of these, the genus *Cutibacterium*, although associated with inflammation, may be involved in maintaining microenvironmental homeostasis in healthy states (7). The healthy bladder microbiome is dominated by Lactobacillus (8), while Cutibacterium and Escherichia are more prominent in the prostate. The urethral microbiome often contains Prevotella, Streptococcus, and so forth, whereas the prostate has lower microbial diversity and is more susceptible to local immune status (7). Anatomical proximity exists between the flora in the urine of healthy men (e.g., *Corynebacterium*, *Streptococcus*) and the prostate microbiome, but the flora of the prostate is more tissue specific (7, 9).

### 2.2 Microbiome differences between prostate cancer tissues and paracancerous tissues

Bacterial genera enriched in prostate cancer tissues: pro-inflammatory and pathogenic: cancer tissues are significantly enriched in *Fusobacterium* (pro-cancer activity), *Streptococcus* (e.g., *S. anginosus*), *Anaerococcus*, and *Propionibacterium acnes* (associated with chronic inflammation) (5). Opportunistic pathogens such as Actinomyces spp. (associated with urinary infections) and Varibaculum cambriense (associated with genital tract infections) are elevated in cancer tissue (10).

Missing flora: genera more commonly found in paraneoplastic or benign tissues include *Pseudomonas* (more abundant in noncancerous tissues) and certain commensal bacteria (e.g., *Curvibacter*) (7).

Changes in microbial diversity: α-diversity: cancerous tissues usually have a lower Shannon’s index than paracancerous tissues, suggesting a decrease in the variety of flora (11). β-diversity: the microbial community structure of cancerous and non-cancerous tissues is significantly different, suggesting selective pressure on the flora in the local microenvironment (12).

Correlation with tumor grading and staging: high Gleason score: gut microbes such as *Akkermansia muciniphila* are enriched in high-risk patients (13). The abundance of *Cutibacterium* and *Staphylococcus* in prostate tissues showed positive correlation with tumor aggressiveness (10).

Inflammation and signaling pathways: activation of inflammation-related pathways such as IL6, STAT3, and NF-κB in cancer tissues correlates with the presence of specific flora (e.g., *Proteobacteria*), which may promote tumor progression through ROS and DNA damage (11, 14).

## 3 Mechanisms of the microbiome in the development of prostate cancer

### 3.1 Inflammatory response and chronic microenvironment

The microbiome can activate pro-inflammatory signaling pathways (e.g., NF-κB, STAT3) through metabolites (e.g., lipopolysaccharide LPS), inducing chronic inflammation and oxidative stress, leading to DNA damage and carcinogenesis. For example, infection of the prostate by pathogens such as *Escherichia coli* (*E. coli*) triggers chronic prostatitis, which disrupts the epithelial barrier, recruits inflammatory cells, and releases reactive oxygen species (ROS), leading to prostate proliferative inflammatory atrophy and prostate intraepithelial neoplasia, which are thought to be precursor states of PCa (6). Pseudomonas promotes BPH by activating the NF-κB signaling pathway, indirectly increasing the risk of cancer (14). LPS produced by intestinal microorganisms enhances prostate cancer cell survival and metastasis by activating the NF-κB pathway through the TLR4 receptor (15).

### 3.2 Immune microenvironment regulation

Furthermore, the microbiome modulates PCa progression by altering tumor-associated immune cell function and the immune microenvironment.

Imbalance of T-cell subsets: in a prostatitis model, Propionibacterium acnes infection leads to an imbalance in the Treg/Th17 cell ratio, which can promote the formation of an inflammatory microenvironment (11).

Immune checkpoint modulation: specific flora (e.g., Bifidobacterium bifidum Bifidobacterium) enhances the efficacy of PD-1/PD-L1 inhibitors, whereas Akkermansia muciniphila is enriched in patients with desmoplasia-resistant prostate cancer and may improve the therapeutic response by modulating the immune response (8, 15, 16).

Macrophage polarization: a dysregulated microbiome promotes the conversion of tumor-associated macrophages (TAMs) to a cancer-promoting phenotype (M2 type) and suppresses anti-tumor immunity (17).

### 3.3 Metabolites and hormone regulation

Gut and prostate microbes can also influence hormone balance and tumor metabolism through metabolites, for example:

Androgen Metabolism: Gut microbes modulate 5α-reductase activity and influence dihydrotestosterone (DHT) levels, which in turn promote PCa progression. After androgen deprivation therapy (ADT), gut flora (e.g., Ruminococcaceae) may contribute to endocrine therapy resistance via steroid metabolic pathways and thus (15).

Short Chain Fatty Acids (SCFAs): SCFAs such as butyric acid produced by some probiotics have anti-inflammatory effects, but under certain conditions may also have an impact on PCa disease progression and regression through epigenetic modifications that promote oncogene expression (16).

Polyamines and bile acids: microbial-derived polyamines (e.g., putrescine) and secondary bile acids (e.g., deoxycholic acid) can also induce prostate cell proliferation and DNA damage, as demonstrated in studies by Garbas (18).

### 3.4 Genotoxicity and epigenetic regulation

Certain microorganisms promote carcinogenesis directly through genotoxic or epigenetic mechanisms:

Genotoxic metabolites: it has been indicated that colibactin produced by *E. coli* induces DNA double-strand breaks and chromosomal instability, which can be directly driven to cause cancerous changes in prostate cells.

Epigenetic regulation: microorganisms can influence the expression of key genes such as PTEN and MYC by secreting miRNAs or regulating host miRNAs (e.g., miR-21, miR-155), which promote cell proliferation and apoptosis resistance and thus have an impact on prostate cancer regression (2).

Viral integration: polyomaviruses (e.g., BK virus) infecting prostate cells may also affect the PCa process by integrating the viral genome to activate oncogenic signaling pathways (e.g., NF-κB) (3).

## 4 Microbiome in prostate cancer diagnosis and treatment

### 4.1 Diagnostic markers

Microbiological characterization of urine/prostate fluid: several studies have shown significant differences in the urine microbiome of PCa patients, such as elevated Enterococcus abundance and enrichment of hypoxia-tolerant bacteria (e.g., Propionibacterium acnes), which can be used as a non-invasive diagnostic tool (5, 18, 19). Meanwhile, some scholars have pointed out that the urine microbiome correlates with tumor markers (e.g., PSA) and clinical staging, and can be detected with high sensitivity by 16S rRNA sequencing, thus accomplishing high-precision auxiliary diagnosis in the early stage of disease (20).

Combined metabolomic analysis: urine metabolomics can improve diagnostic specificity by detecting levels of metabolites such as creatine and citrate. For example, it has been emphasized that the combination of metabolic markers and microbiome profiling can more accurately differentiate PCa from benign prostatic hyperplasia (BPH), thus reducing overdiagnosis (21, 22). In addition, clinical studies have shown that metabolomic modeling can be effective in assessing response to endocrine therapy (23).

### 4.2 Prognostic assessment and outcome prediction

Relationship between microbiome diversity index and survival (high diversity predicts better prognosis).

Gut microbial characterization predicts radiotherapy/chemotherapy sensitivity (e.g., *Faecalibacterium* vs. desmoplasia-resistant prostate cancer).

Microbiome diversity index: high diversity in the gut and urine microbiomes is usually associated with a better prognosis. Some researchers have suggested that dysbiosis of the gut flora (e.g., Bacteroides and Streptococcus enrichment) may promote tumor progression and that patients with high diversity are more sensitive to chemotherapy or radiotherapy (6, 24). However, some researchers have also suggested that some studies did not find significant differences in gut microbial diversity between cancer and control groups, suggesting that further validation of the microbiome’s relationship between cancer and control groups is needed (5).

Specific flora and therapeutic sensitivity: for example, reduced abundance of Faecalibacterium has been associated with desmoplasia-resistant prostate cancer, whereas Akkermansia muciniphila may influence the efficacy of radiotherapy by modulating the inflammatory microenvironment (25). It has also been mentioned that gut flora characteristics may predict side effects and resistance to ADT (15).

### 4.3 Treatment strategies

Probiotic/prebiotic interventions: the side effects of ADT (e.g., metabolic disorders and immunosuppression) can be ameliorated by regulating the intestinal flora. Some researchers have shown in their treatises that supplementation with probiotics (e.g., Lactobacillus) or dietary fiber restores the balance of the flora and can enhance anti-tumor immunity (15, 24).

Antibiotic-targeted therapy: removal of cancer-promoting bacteria (e.g., Fusobacterium nucleatum, Propionibacterium acnes) inhibits chronic inflammation and tumor microenvironment activation. It has been demonstrated in studies that antibiotics combined with chemotherapy can reduce the risk of PCa recurrence (6, 26).

Combined immunotherapy: microbiome modulation may enhance the effects of immunotherapy. For example, some scholars have noted in their studies that the gut flora influences immune checkpoint inhibitors (e.g., PD-1 inhibition (24, 27). through the NF-κB-IL6-STAT3 axis.

## 5 Challenges and prospects

Technical bottlenecks: sequencing accuracy of low biomass samples, low sample size of prostate tissue, risk of PCR amplification bias, and chimera with conventional 16S rRNA sequencing, leading to incomplete taxonomic information (11). Metagenomic sequencing avoids amplification bias but is limited by incomplete databases (e.g., fungal genome annotations) (25) and high detection thresholds for low-abundance microbes (28). Long read-length sequencing technologies (e.g., PacBio, Oxford Nanopore) and single-cell macrogenomics can improve assembly quality (29). Also, standardized sample processing procedures (e.g., DNA extraction methods) can help reduce variability (30).

Causal mechanism validation models, Organoid/Humanized Mouse Models Insufficient Physiological Relevance in Simulating Cellular Communication and Metabolic Activity (31). It has also been pointed out that a combination of functional metagenomics (e.g., metabolomics) and *in vitro* culture models (e.g., HuMiX) is needed to reveal the mechanisms of microbe-host interactions (27, 32). In addition, the finding that symbiotic bacteria promote endocrine resistance through androgen synthesis has been suggested to suggest the need to optimize models to capture the dynamic effects of microbes on hormonal pathways (33).

### 5.1 Clinical translation

Large-scale cohort validation of markers, sample heterogeneity in current microbial marker studies (e.g., use of paracancerous tissues as controls) (25), the need to include healthy controls and to control for confounders such as ethnicity, diet, and so forth (34, 35). There is also an emphasis on standardizing data collection and analysis processes (e.g., central logarithmic ratio transformation for constitutive data), combined with machine learning to identify robust microbial community profiles (36).

Personalized flora transplantation protocols: fecal microbial transplantation (FMT) has been successful in C. difficile infections (37), but the field of PCa needs to address donor-recipient matching, the impact of non-bacterial microbes (e.g., fungi, phages) (25, 38). Combining engineered microbes (e.g., CRISPR phages) to target drug-resistant bacteria has been suggested (25, 39) and monitoring metabolic and immune responses after transplantation through multi-omics (32).

Despite the fact that tumors share a host immune microenvironment with paraneoplastic tissues, fewer transcriptomic studies have been conducted on noncancerous BPH tissues, and further exploration of their differences from PCa is needed in the future. Current research suggests that microbial profiles may be influenced by geography, diet, and other factors, but direct evidence on racial differences is limited and needs to be validated in larger cohort studies.

### 5.2 Future directions

#### 5.2.1 Bidirectional regulation of the “gut-prostate axis”

Gut flora regulates systemic inflammation and hormone levels through metabolites (e.g., short-chain fatty acids), while localized prostate microbes (e.g., *Cutibacterium*) may exacerbate cancer progression through androgen metabolism (17, 33). For example, gut-derived short-chain fatty acids (SCFAs) may suppress inflammation via G-protein–coupled receptors, while *Cutibacterium* in the prostate may convert testosterone to dihydrotestosterone, promoting tumor growth (17). It has also been proposed to study microbiome-HPA axis interactions, especially the impact of the stress response on the prostate microenvironment (35), which may require further integration of metabolomics to track tryptophan/lipid metabolic pathways (34).

#### 5.2.2 Cross-omics integration and multidimensional networks

Multi-omics (macro-genome + metabolome + proteome) can reveal associations between microbial functional activities and host phenotypes (32), for example, deep learning integration of genomic mutation and metabolic reprogramming data can predict PCa recurrence (33). The development of unified databases (e.g., KEGG, Pfam) to standardize functional annotations and the use of tools such as iPath to visualize metabolic pathway interactions can also be strengthened to further enhance cross-academic group integration and multidimensional network linkages (39).

#### 5.2.3 Impact of race and lifestyle

Diet (e.g., high-fat diets) and antibiotic use can significantly alter the gut flora structure (37), potentially influencing PCa risk through the “microbial-immune-metabolic” axis. The need to incorporate ethnically stratified analyses in cohort design to identify specific microbial markers has been noted in the study (34). In addition, further attention to the dynamic balance of fungal-bacterial interactions in immunosuppressed patients is also important to study PCa regression (25).
